# Controlling Indomethacin Release through Vapor-Phase Deposited Hydrogel Films by Adjusting the Cross-linker Density

**DOI:** 10.1038/s41598-018-24238-w

**Published:** 2018-05-08

**Authors:** Paul Christian, Stephan Tumphart, Heike M. A. Ehmann, Hans Riegler, Anna Maria Coclite, Oliver Werzer

**Affiliations:** 1 Institute for Solid State Physics, NAWI Graz, Graz University of Technology, 8010 Graz, Austria; 2 Institute of Pharmaceutical Sciences, Department of Pharmaceutical Technology, University of Graz, 8010 Graz, Austria

## Abstract

Vapor-phase deposited polymer coatings are applied on thin indomethacin films to modify the drug release. Hydrogel-forming co-polymers of 2-hydroxyethyl methacrylate and ethylene glycol dimethacrylate were prepared directly on top of solution cast indomethacin thin films by initiated Chemical Vapor Deposition (iCVD). This technique allows for solvent-free processing under mild conditions, thus minimizing a potential impact on the pharmaceutical. The drug release behavior, among other properties, was evaluated for polymers of different compositions and at different temperatures. The data show that the release kinetics can be tuned by several orders of magnitude as the cross-linker fraction is varied in the polymer coating. While uncoated indomethacin films were fully released within an hour, polymer coatings showed gradual liberation over several hours to days. Additional insight is gained from evaluating the experimental dissolution data in the framework of diffusive transport. The results of this study show that the iCVD technique has some promises for pharmaceutical technology, potentially allowing for tailored release behavior also for other drug systems.

## Introduction

**Figure 1:**
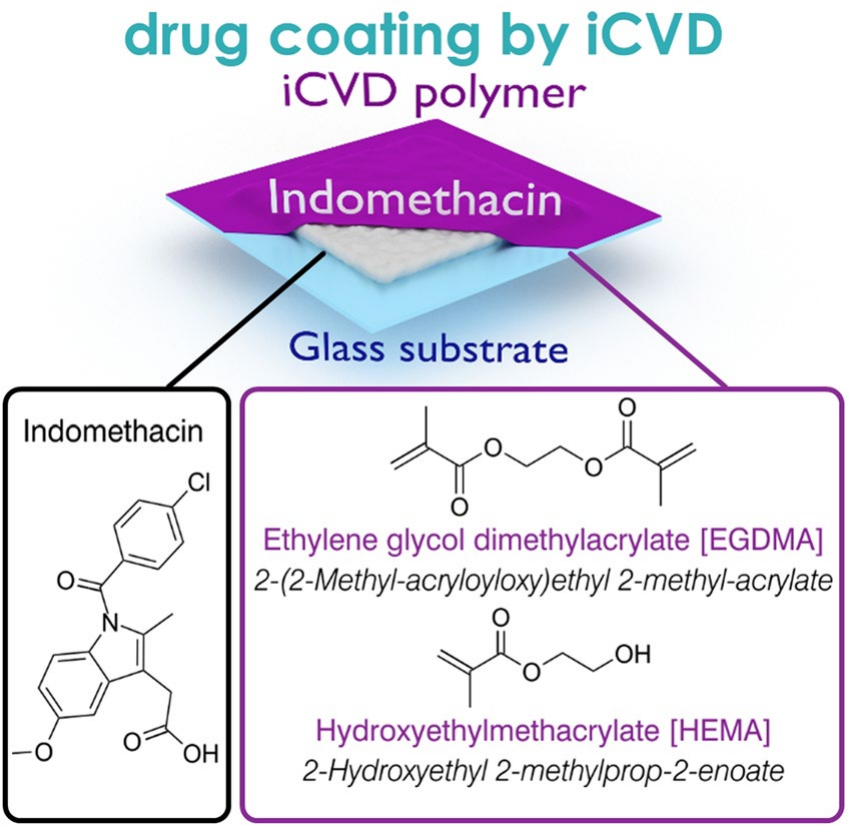
Cutaway drawing of the sample structure, depicting indomethacin drug layers on glass substrates, encapsulated by an iCVD polymer layer (top left). At the bottom, structural formulas of indomethacin and of the iCVD monomers, labeled by their trivial names, are provided. The corresponding abbreviations are stated in square brackets, the IUPAC names in italic letters.

To enhance efficiency of existing medication and to develop new therapeutic options for currently untreatable medical conditions, pharmaceutical research focuses not only on the screening of new active pharmaceutical ingredients (APIs) but also on the exploration of different administration routes and alternative dosage forms. Polymers can assume various crucial functions in drug formulations, providing protection against premature release^1,2^, stabilizing specific solid state forms^3,4^, enabling site-specific drug activity^5,6^ or being functionalized themselves by anchoring drugs or proteins directly to their structure (*polymer therapeutics*)^7^. The drug release from such polymer systems is usually facilitated by either degradation/erosion of the polymer host or by diffusion of the drug; for the latter mechanism, matrix-, reservoir- and hydrogel-based systems are usually differentiated^8^. In matrix systems, the API is homogenously dispersed inside a suitable host material such as cellulose^9^ or lipids^10^, with the tortuosity of the material determining release kinetics^11^. While hydrogel systems can also be considered matrix systems as they are usually loaded with an API, drug release occurs upon swelling of the hydrogel and is governed by the mesh size of the polymer^12^. In reservoir systems, on the other hand, a core-shell structure is usually present, with the API being encapsulated by an outer membrane; as such devices can store and then gradually release a larger amount of the drug, they are suitable for internal application as implants^13^. Reservoir systems have mostly been investigated for conventional drug forms such as pellets^14,15^, while less work has been performed on coatings of thin film dosage forms by solution-based methods. However, the preparation of thin pharmaceutical films is of particular interest; dosage forms like patches can allow for perpetual therapeutic action while being easy to apply/remove^16^. When combined with sequential polymer deposition, multilayer devices with different functionalities can even be realized^17,18^. However, preparing defined and well-separated layers of API and polymers can be quite challenging, especially in the case of thin films. Solution-based techniques commonly applied in the preparation of polymer layers will usually affect the drug as well. While this might not be a limitation to matrix-type systems where intermixing is desired, such an approach is not well suited for reservoir systems. Even if intermixing is avoided by use of orthogonal solvents, other solvent-drug interactions may still be present which in turn can change the solid-state of the drug. In addition, environmental and health issues might limit excessive use of solvents and residual solvents have to be avoided in the final films. A solvent-free approach lies in polymer synthesis by initiated Chemical Vapor Deposition (iCVD)^19^. This technique allows for the synthesis of polymeric layers directly at an interface while achieving conformality and defined chemical composition even on delicate substrates^20,21^. The iCVD process is based on the mechanism of radical polymerization in solution, with the difference being that all reagents are supplied in their vapor phase form at low reactor pressure (usually held between 10 to 100 Pa). An initiator, usually a peroxide with a labile oxygen-oxygen bond, decomposes thermally at a heated filament (typically 200–300 °C), thus forming radicals. These radicals can then interact with vinyl bonds of monomer units absorbed on the substrate surface, forming a monomer-radical complex. Additional monomer units then attach, facilitating polymer chain growth until being terminated by either another radical or monomer-radical complex. A more detailed description about iCVD in general and the associated mechanisms can be found in literature^22,23^. Previous applications of iCVD polymers in the field of biomedicine comprise the fabrication of thermo- and pH-responsive drug delivery systems^24–26^. In these cases, delivery was mostly studied in relation to the stimuli-responsive properties of the iCVD coatings but little focus was put on the release kinetics. The latter is of great importance for pharmaceutical dosing, where direct control can enable therapies that are specific to the individual and the situation. For this reason, the present study focuses on how variations in polymer mesh size affect the drug release behavior. Polymers of 2-hydroxyethyl methacrylate (HEMA) and ethylene glycol dimethacrylate (EGDMA) were deposited by iCVD on top of indomethacin thin films (for the structural formulas and an illustration of the sample structure, see Fig. 1). Hydrogel-forming polymers based on HEMA are widely studied as drug release platforms in matrix systems^27–29^. This makes HEMA also interesting in the present application as a coating layer for reservoir drug systems; the hydrophilic character facilitates swelling in aqueous environment, which in turn should foster drug release (compared to release through the polymer coating in an unswollen state). The addition of EGDMA, a cross-linker, enhances the mechanical stability of the coatings and can be used to control the hydrophilic/-phobic character of the material (and thus, the mesh size and water uptake). The impact of the iCVD coating on the solid-state, the morphology and the dissolution behavior of the indomethacin films is investigated as function of polymer composition and dissolution media temperature. While amorphous drug formulations can be desirable in applications where enhanced drug release is favorable, this will often result in additional polymer-drug interactions. For instance, polymeric coatings exhibit strong wrinkling when deposited on amorphous API films while such behavior is absent on crystalline films due to a smaller difference in elastic modulus^30^. Also, polymeric coatings can influence solid state transitions of drug layers, both enhancing or retarding crystallization kinetics depending on the drug-polymer interactions^31^. To minimize these and other influences, the focus of the present study is put on polymer encapsulation of crystalline indomethacin films.

## Results

### Indomethacin film characterization

**Figure 2:**
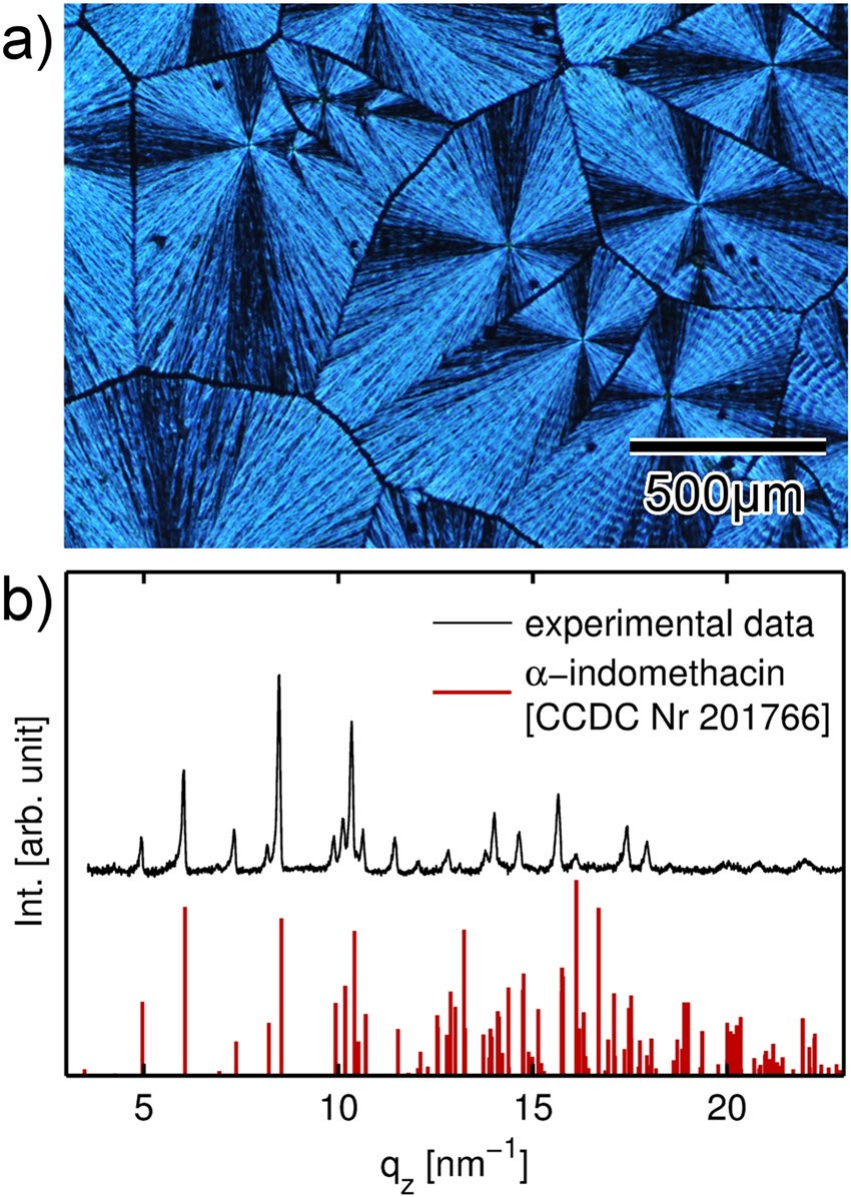
(**a**) Optical microscopy image of an indomethacin sample in the crystalline state after ethanol solvent vapor annealing. The image was taken under crossed polarizers. (**b**) Specular X-ray diffraction pattern of a solvent-annealed sample and theoretical powder diffraction pattern of the indomethacin α-polymorph. Please note that experimental data are baseline corrected to remove scattering from the amorphous glass substrate.

Homogenous indomethacin films were obtained by solution casting. Initially, a uniform liquid layer forms on the substrate due to the good wetting properties of THF on glass surfaces. Upon fast solvent removal, homogenous indomethacin films form. The solid state of such films is amorphous and crystalline fractions are neither noted in the optical micrographs nor (more qualitatively) in X-ray diffraction patterns (*cf*. to Figure S1). As film formation occurs quickly due to the high vapor pressure of THF, this leaves little to no time to the individual molecules to adapt an ordered (long range) arrangement, thus the system remains in a meta-stable amorphous state. While samples will transfer to a crystalline state eventually, neither storage at ambient conditions nor at 50 °C for five consecutive days did change the solid state. Indeed, previous studies on amorphous indomethacin have found induction times between 15 to 50 days when employing temperatures below 60 °C^32^. However, by casting indomethacin from different solvents or by artificially reducing the solvent evaporation rate (e.g. by covering the sample with a Petri dish), the formation of some crystalline fraction results, which grows into larger domains within a few hours. In addition, some solvents yield distinct crystal structures (polymorphs); for instance, indomethacin crystallizes from ethanol solution in the α-form (monoclinic)^33^ while the γ-form (triclinic) is obtained from ethyl ether^34^. Similarly, this solvent-specific behavior can also be used to transfer amorphous films into specific crystal forms. In this study, ethanol solvent vapor annealing was used for controlled crystallization. Within 48 hours, samples exposed to ethanol vapor in a sealed container had fully crystallized. Figure 2a shows an optical microscopy image of such a sample taken under crossed polarizers, revealing Maltese cross patterns characteristic for spherulitic growth (the patterns are due to birefringence). The size of a single spherulitic domain varies from approximately 200 to 850 µm. The crystalline nature of such films is also confirmed by X-ray diffraction (see Fig. 2b). The specular diffraction pattern exhibits multiple Bragg peaks and the indexation reveals the presence of the indomethacin α-form^35^ (CCDC Nr 201766). The various Bragg peaks present in the pattern indicate that a preferred orientation is absent, i.e. the individual spherulitic branches are also rotated in respect to the substrate normal and the crystallites exhibit a powder-like distribution.

### Polymer deposition onto crystalline drug layers

**Figure 3:**
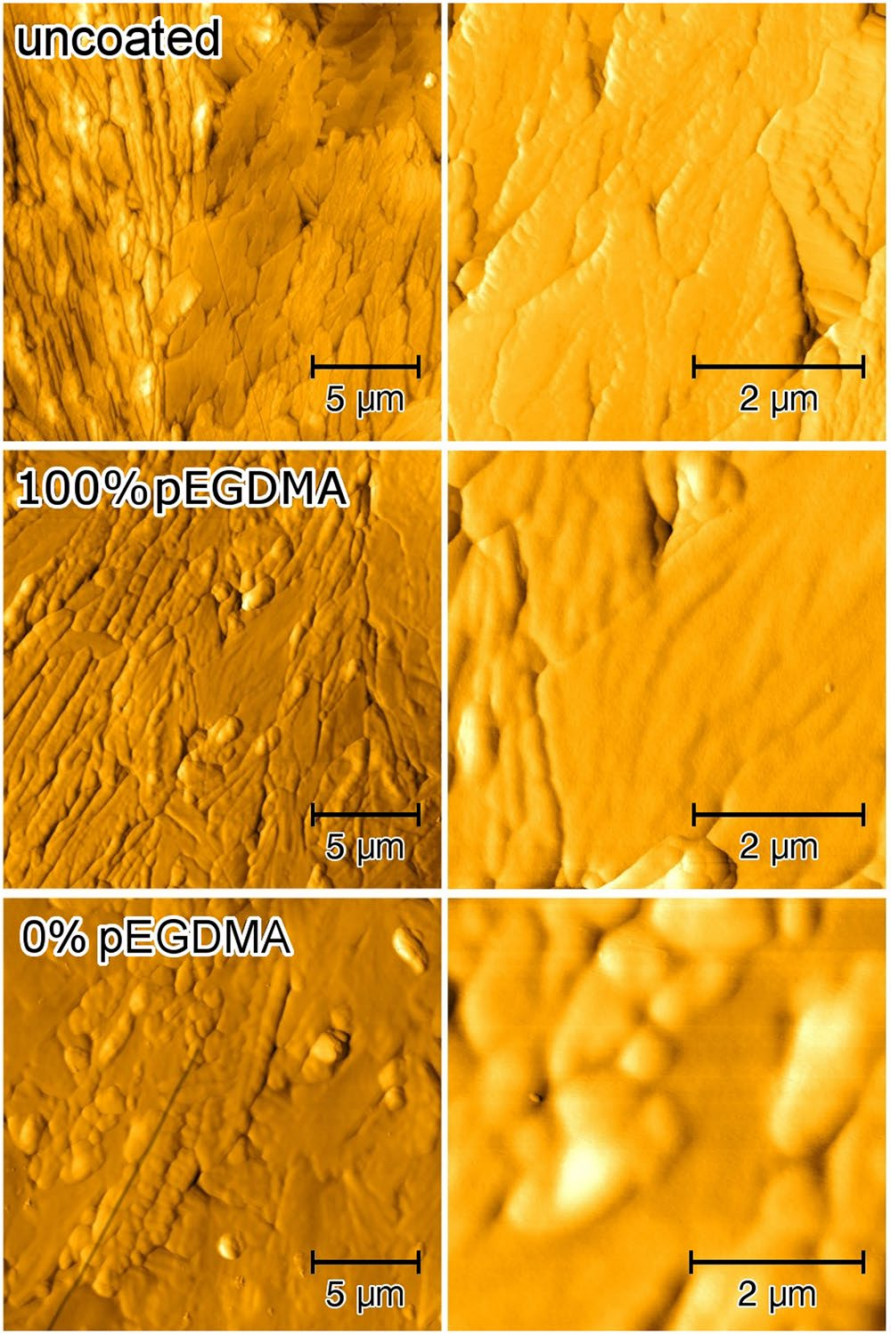
AFM micrographs of an uncoated, crystalline indomethacin sample (top) and of samples coated with either a pEGDMA (center) or a p(HEMA-*co*-EGDMA) polymer layer (bottom). The coating thicknesses are approximately 200 nm. Left column depicts the samples at larger scales, for details refer to the right column.

The coating of crystalline indomethacin with iCVD polymers leaves the underlying drug layer mostly unaffected and changes in morphology or solid state are not observed. Spherulitic indomethacin domains dominate the optical appearance as the polymer coatings are optically transparent. Thus, samples were investigated by AFM, the data being summarized in Fig. 3. The uncoated surface exhibits branched, feather-like structures typical for the spherulitic growth of indomethacin crystals. These structures are simply replicated by the polymer coatings grown atop, underlining the conformity of iCVD coatings. Also, changes in the solid state were not detected from XRD scans (*cf*. Figure S2), indicating the gentle nature of the iCVD process.

A more detailed look at the data in Fig. 3 reveals that surface features appear *smoothed* when covered by the polymers, indicating that such structures become slowly buried as the polymer thickness increases during deposition. While this behavior is noted both for the pEGDMA homopolymer and the hydrogel-forming p(HEMA-*co*-EGDMA) coating, detailed inspection reveals another difference. Graphs of the latter depict a *blurred* surface, more reminiscent to a measurement artifact than to actual morphology. Likely, this is caused by a softening of the polymer when taking up water from the environment (approx. 25 °C, 70% humidity), thus forming a hydrogel^36^. Under dry conditions, the non-polar methyl groups are oriented towards the environment-polymer interface while the polar hydroxyethyl are turned inward, with a reversed behavior being observed in the swollen state, thus increasing flexibility^37^.

### Characterization of iCVD coatings on bare substrates

The deposition of p(HEMA-*co*-EGDMA) layers onto silica substrates replicates the surface so that morphologies similar to a bare substrate result (Figure S3). The chemical composition of the polymers had no impact on the morphology of the films. The polymer layers were prepared at a thickness of about 200 nm. This choice was made based on pre-evaluations and provides the best compromise for the iCVD setup in use in terms of preparation times (deposition rates were below 10 nm/min) and the final coating performance. All deposited polymers were subject to chemical characterization by infrared spectroscopy, allowing the HEMA to EGDMA ratios to be determined (data are shown in Figure S4). Three different compositions are exemplarily considered for the dissolution experiments, i.e. the ones with 25%, 50% and 100% EGDMA. It should be noted that with no or little cross-linking, pHEMA exhibits strong water uptake, thus detaching easily from the surface. While such a behavior is undesirable for controlled release, it is potentially useful for targeted drug delivery, especially when combined with stimuli-responsive functionalities.

**Figure 4:**
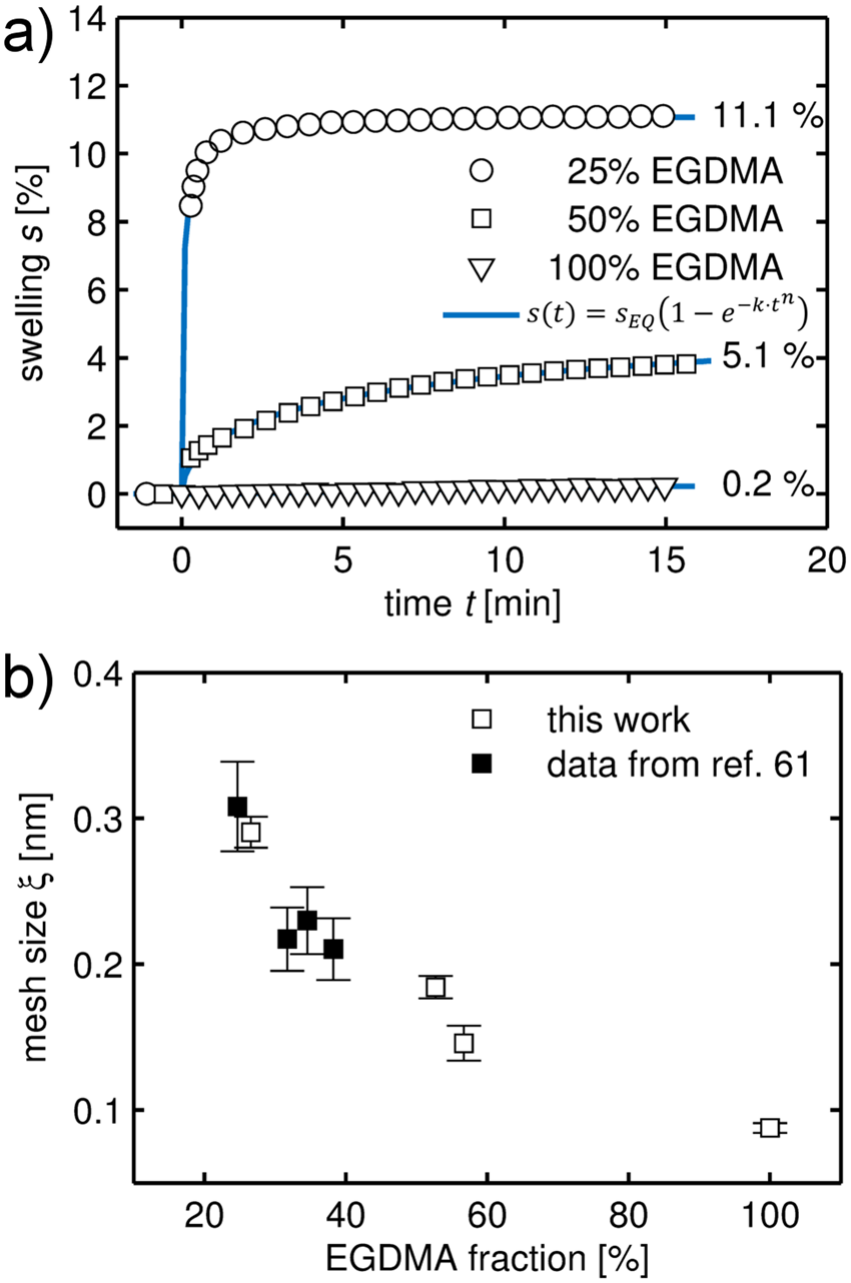
(**a**) Swelling ratios of p(HEMA-*co*-EGDMA) films with different EGDMA volume fractions (100% refers to the homopolymer) as a function of time, as determined from ellipsometric measurements. Data are fitted by the empirical model provided in Eq. (1) and labeled with the values of equilibrium swelling *s*_*EQ*_. (**b**) Polymer mesh size as a function of EGDMA cross-linker content, calculated from the equilibrium swelling ratio according to Eq. (3). Data from the present study are plotted with open symbols (□), data from a previous publication^61^ are represented by filled symbols (■).

The ability of HEMA copolymers to swell in aqueous environment is crucial for the drug release; uptake of water (or any other liquid) depends strongly on the mesh size of the polymer, which will also strongly influence how drug transport proceeds through the membrane. To evaluate the swellability of the studied polymers, polymer film thickness was monitored during aqueous swelling by *in situ* ellipsometry. While drug release studies were performed in a phosphate buffer solution instead of *pure* water, a comparable polymer swelling behavior is observed for both media (*cf*. Figure S5). In Fig. 4a, exemplary swelling curves are depicted for different polymer compositions. The data evidence how swelling is drastically decreased as the cross-linker fraction is increased in the polymer. While a polymer with 25% EGDMA content exhibits swelling up to 11%, the EGDMA homopolymer shows a negligible thickness increase when immersed into water. The hydrophobic character of EGDMA paired with strong cross-linking (and physical entanglement) in the polymer prevents water penetration at large so that only imperfections (such as unpolymerized vinyl groups or pinholes) should contribute to water uptake. From the equilibrium swelling degree *s*_*EQ*_, the mesh size of such polymers can be estimated. For this, the evolution of swelling degree $s=\frac{({\rm{\Delta }}d)}{({d}_{0})}$ as a function of time is fitted to an empirical model by

1 $$s(t)={s}_{EQ}(1-{e}^{-k\cdot {t}^{n}}),$$

with *d*_0_ being the initial (unswollen) polymer thickness, *k* being the rate constant and *n* being an empirical exponent. This equation is a simple sigmoid function and allows for a more reliable estimation of *s*_*EQ*_ in cases where equilibrium is not reached within the time of the experiment. The average molecular weight between cross-links $\overline{{M}_{c}}$ in the layer is related to the polymer volume fraction at equilibrium $\varphi =\frac{({d}_{0})}{({d}_{0}+{\rm{\Delta }}d)}=\frac{1}{{s}_{EQ}+1}$ by

2 $$\overline{{M}_{c}}=-\,{V}_{s}{\rho }_{pol}\frac{(\frac{1}{\varphi }-\frac{\varphi }{2})}{\,\mathrm{ln}(1-\varphi )+\varphi +\chi {\varphi }^{2}}\,,$$

where *V*_*s*_ denotes the molar volume of the solvent (*V*_*H*2*O*_ = 18.03 cm^3^/mol), *ρ*_*pol*_ is the polymer density (for pHEMA^38^, *ρ*_*HEMA*_ = 1.274 g/cm^3^) and *χ* is the polymer-solvent interaction parameter. For pHEMA, this interaction parameter was found to be dependent on *ϕ*, with *χ* = 0.320 + 0.904*ϕ*^39^. Equation (2) is a modified form of the *Flory-Rehner* equation^40,41^, accounting for the case of a surface-attached polymer which is thus restricted to one dimensional swelling^42^. A full derivation of this expression is provided in the supplementary information. The mesh size *ξ* is then given by

3 $$\xi =l{\varphi }^{-1/3}{C}_{n}^{1/2}{(\frac{2\bar{{M}_{c}}}{{M}_{m}})}^{1/2},$$

with *l* refering to the characteristic atomic bond length of the backbone (for C-C, *l* = 0.154 nm) and *C*_*n*_ denoting the Flory characteristic ratio (rigidity factor). For pHEMA, literature suggests *C*_*n*_ = (6.9 ± 0.5).

Evaluating experimental swelling data of polymers with different fractions of cross-linker in the framework of Eq. (3) yields mesh sizes ranging from (0.29 ± 0.01) nm for 25% EGDMA to (0.15 ± 0.01) nm for 57% EGDMA (see Fig. 4b). For pEGDMA, a theoretical mesh size of (0.088 ± 0.004) nm is calculated. Given that the diameter of water is about 0.27 nm^43^, this raises the question how such values have to be interpreted. The theoretical analysis works on the premise of a defect-free, syndiotactic polymer where the local structure is repeated indefinitely throughout the swelling film. In praxis, polymers will often be (at least partially) atatic, displaying physical entanglement and incomplete vinyl conversion, among other defects, so that the mesh size will only be a mean measure of a distributed quantity. This is also evident when comparing the average number of links $N=2\overline{{M}_{c}}/{M}_{m}$, determined from the swelling studies, to the (theoretical) ratio calculated from the volume fractions, *N*_*theory*_ = *F*_*HEMA*_/*F*_*EGDMA*_^44^. For instance, *N*_*theory*_ calculates to 2.7 for the sample containing 25% EGDMA, while the swelling studies determine a value of just 0.5, meaning that the polymer appears (in average) stronger cross-linked than expected from just the composition. This difference is usually attributed to physical entanglement, which is limiting the overall swelling^44^.

### Indomethacin dissolution without coating

The dissolution behavior of uncoated indomethacin films was studied in a pH 5.8 phosphate buffer solution held at 25 °C. As indomethacin is an ionizable drug, its solubility varies with the species and the pH value of the buffer solution^45^. For the phosphate buffer used, indomethacin solubility at pH 5.8 and 25 °C is about 0.1 mg/ml (see also the Supporting Information)^46^. This means that the tested samples (containing about 2 mg of the drug) can fully be dissolved in the volume of the dissolution medium (50 mL).

**Figure 5:**
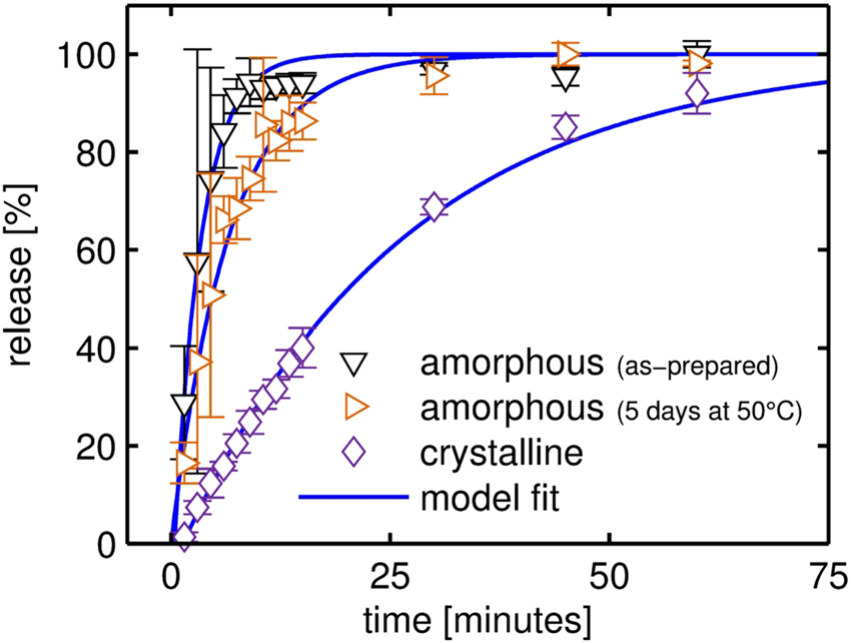
Drug release performance of thin indomethacin films in the amorphous (as-prepared and after storage) and the crystalline state. All data has been collected at 25 °C. A detailed description of the model fit is provided in the text (section *data modeling*).

Both amorphous and crystalline indomethacin samples were tested, with the individual release profiles being depicted in Fig. 5. For an as-prepared, amorphous sample, rapid release kinetics are observed. Initially, drug release from the substrate surface proceeds at a constant rate, the slope being 19%/min. As the released volume fraction surpasses sixty percent, a gradual decrease of the release rate results (elimination phase), until full dissolution is achieved after 30 minutes. In general, dissolution of thin drug layers from a surfaces proceeds at a very fast pace as the surface area per volume, which is directly proportional to the release kinetic, is very large^47,48^. Such a rapid release might be desired in cases where immediate therapeutic action is required. For comparison, amorphous samples were also tested after being stored at 50 °C under ambient atmosphere for five consecutive days. This does not change, at least in the limit of detection, the sample properties (i.e. morphology, solid state). Similar to the as-prepared samples, dissolution experiments reveal a rapid release, again displaying a constant rate at first. The slope of 11%/min in the initial release means that indomethacin dissolution is a bit slower in this case and full release results after approximately 45 minutes. While the exact reason for this difference cannot be unambiguously identified within this study, one possible explanation can be amorphous - crystalline phase transitions during dissolution experiments^49^. While such an effect might also be present here, the (overall) fast dissolution rate means that the period for significant crystallization is likely too small. It should also be mentioned that due to the rapid dissolution behavior, error bars are rather large for these data sets. To avoid these issues when testing polymer coated samples, only samples containing crystalline indomethacin were employed.

Crystalline indomethacin, on the other hand, displays a significantly slower drug release when compared to its amorphous counterpart. Full dissolution of crystalline samples takes approximately two hours, meaning that a modification of the solid state form allows already some control over the drug release behavior. The slower drug release is the result of an additional energetic barrier that molecules need to overcome in the dissolution process due to being confined to distinct lattice sites.

It should also be mentioned that it was not possible to gain statistically reasonable results with the applied dissolution measurement strategy when increasing the temperature to 37 °C or even 50 °C as the dissolution rates were too high.

### Dissolution of crystalline indomethacin with iCVD polymer coating

**Figure 6:**
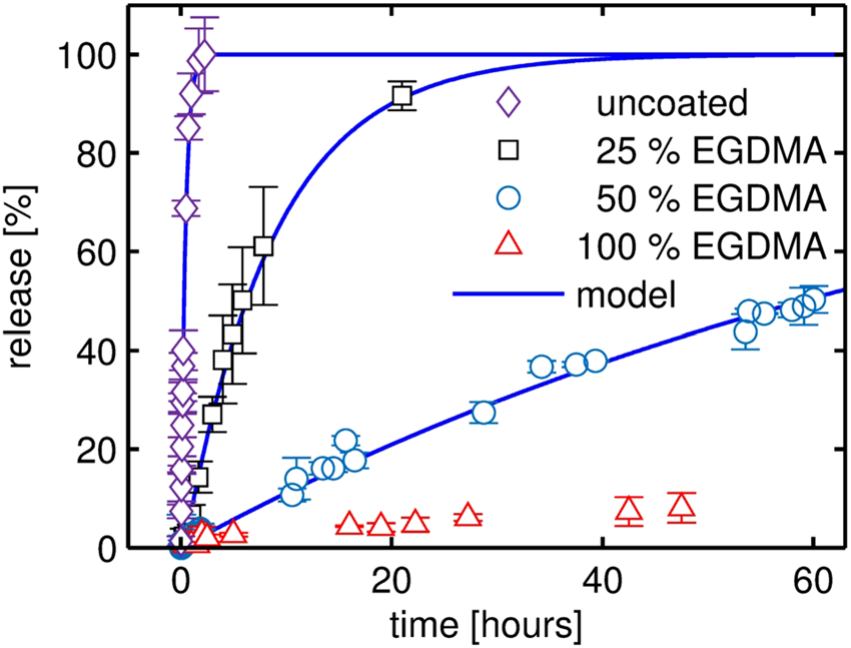
Dissolution of various crystalline indomethacin layers with and without iCVD coatings of different composition. All data were measured at a constant temperature of 25 °C and only the first 60 hours of the dissolution processes are depicted.

In Fig. 6, the dissolution profiles of crystalline indomethacin films, coated with different iCVD polymers, are depicted. The dissolution medium was held at 25 °C and the dissolution profile of the uncoated sample is also shown for comparison. While the release of uncoated indomethacin films from glass substrates proceeds on the time scale of minutes, drug liberation from coated samples occurs over a span of several hours to days. Moreover, distinct release behaviors are noted for the different polymer compositions; as the number of cross-links is increased in the polymer, drug release is increasingly retarded. For example, full indomethacin release takes over 20 hours in the case of a 25% EGDMA coating, prolonging drug release by a factor of 10 compared to the uncoated film. As the hydrogel character of the polymer coating is reduced by increasing EGDMA fraction, release kinetics are slowed down further. Interestingly, some release is also noted from a sample coated with the EGDMA homopolymer, albeit only a fraction of the other samples is liberated in the same time frame. Despite the high cross-linking degree and little water penetration (compare swelling data in Fig. 4), the pEGDMA coating allows for some release. This behavior suggests that coating imperfections are mainly responsible for this kind of release; oligomeric portions, pinholes, edge defects and/or partial ablation of the polymer coating may cause such a *leaking* release.

Therefore, dissolution data for the pEGDMA coating can be regarded as a measure for the integrity and mechanical stability of such coatings. Given that less than 10% of indomethacin is released over the course of 50 hours, a reasonable quality of these films can be expected. This is also in agreement with the fact that morphological investigations did not reveal any obvious imperfections, as mentioned previously. Overall, this means that such coatings are very effective in controlling drug release from the model substrate, here a glass surface. A more detailed analysis of release kinetics as function of temperature for the different coatings is provided in the following sections.

### Temperature dependent release

**Figure 7:**
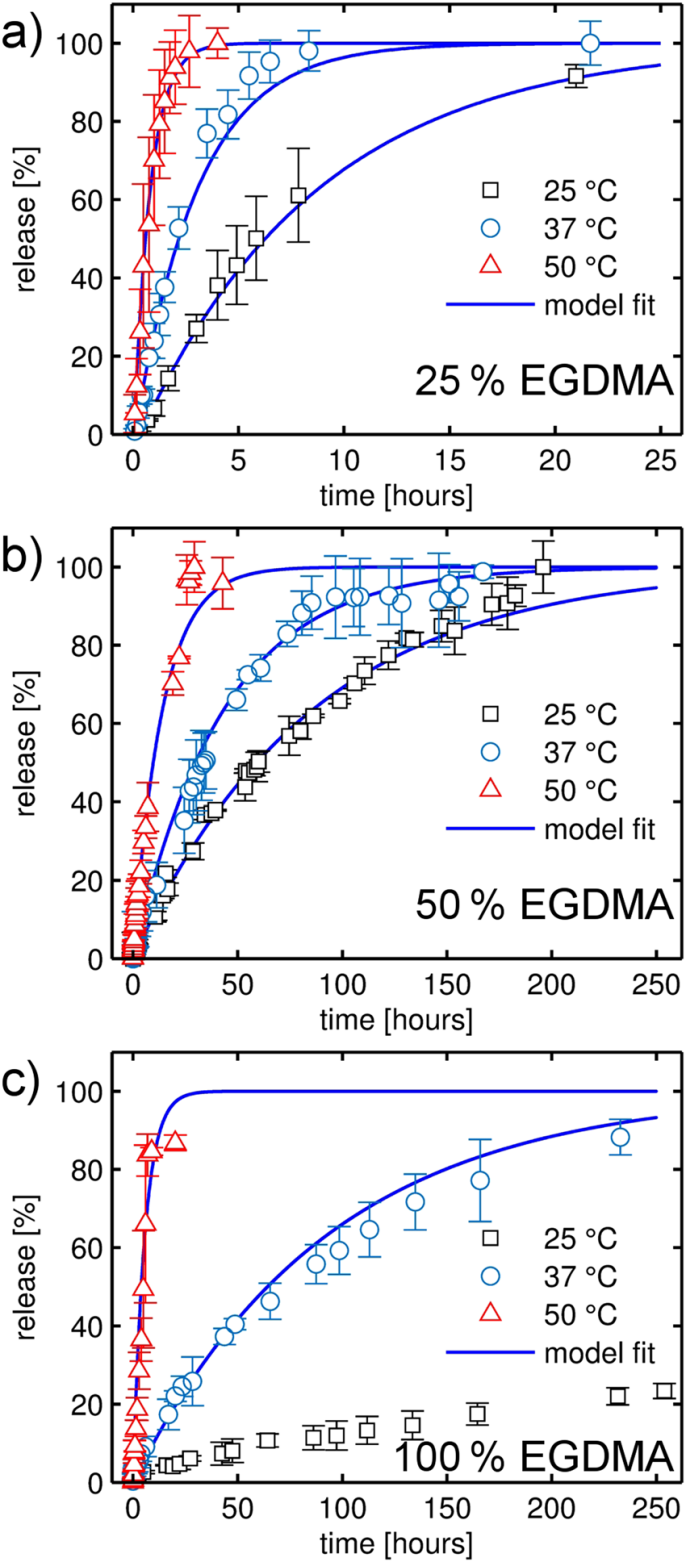
Temperature-dependent dissolution profiles of indomethacin samples coated with different p(HEMA-*co*-EGDMA) films. Release dynamics are depicted at three different temperatures (25, 37 and 50 °C) for polymer coatings consisting either of 25 (**a**), 50 (**b**) or 100% (**c**) EGDMA. Fits to the experimental data by Eq. (5) are indicated by solid, blue lines. Please note that graph (**a**) features a much shorter time frame than graphs (**b**,**c**).

In general, an increase in temperature means that drug diffusion and thus dissolution processes are accelerated. To test this, dissolution measurements were performed at three different temperatures (25, 37 and 50 °C), the data being summarized in Fig. 7. At higher temperatures, faster release kinetics result for all the tested polymer compositions. While higher temperatures lead to significantly accelerated drug liberation, a *burst release* is not apparent from the present data, indicating that the polymer retains its mechanical integrity for at least some time during the dissolution experiment. The polymer coating with the least amount of EGDMA cross-linker exhibits the fastest release kinetics throughout all the tested temperature regimes. For example, by increasing the temperature to 37 °C (approximately human body temperature), full indomethacin release is achieved almost four times faster through a 25% EGDMA coating than at 25 °C. Increasing the temperature further to 50 °C, the necessary timeframe is reduced to 2.5 hours. At a cross-linker ratio of 50%, a similar behavior is observed, though the time scale for the release is increased to days instead of hours (Fig. 7b). Also, the improvement in dissolution rate upon a temperature increase to 37 °C is less pronounced; for full release, the required time is reduced from more than nine days (at 25 °C) to 3.8 days. An increase to 50 °C accelerates the dissolution behavior further and full release is observed after approximately 2 days.

A different behavior is observed in the case of the EGDMA homopolymer (Fig. 7c). At 25 °C, little drug release is observed; even after 10 days, less than a quarter of the total drug loading has been liberated. Increasing the temperature to 37 °C, release kinetics are becoming increasingly similar to the ones recorded for the copolymers. However, full release is again not observed within the time scale of the experiment. As mentioned previously, release in the case of the pEGDMA polymer should be considered being due to polymer imperfections. In particular, increased temperature will cause additional stress between the several interfaces (polymer-drug, polymer-substrate, drug-substrate), which in turn will foster rupture formation and (partial) ablation. This is also evident when looking at the release kinetics at 50 °C; release proceeds even faster than the one of the 50% EGDMA copolymer sample. As the rigidity is increased with the cross-linker content, the difference in elastic modulus between drug and polymer is most pronounced for the EGDMA homopolymer. Further tuning of the iCVD process parameters and polymer properties (composition and comonomer choice) can likely help to improve the stability, if desired.

### Dissolution data modeling

Qualitative differences in the dissolution behavior of uncoated and coated indomethacin samples can be directly noted from the curves in Figs 5 and 7. When a polymeric coating is applied, the drug release rate is significantly reduced, indicating that the coating layer becomes the rate limiting step in the dissolution process. For a more quantitative evaluation, a mathematical model was fitted to the experimental data. Depending on the dissolution mechanism, two main models are typically distinguished: diffusion layer models and interfacial barrier models^50^. In the interfacial barrier model, dissolution is assumed to be limited by the solvation process at the solid-liquid interface. In the diffusion layer model, the rate limiting step is attributed to the diffusive transport of the drug instead. In the present case, drug molecules are transferred from the solid to the solvated state at the drug-polymer interface. As only the polymer composition (i.e. the cross-linker fraction) was varied while maintaining coating thickness and drug dose, differences between samples are attributed to the altered (diffusive) transport through the polymer coatings.

For diffusion-limited drug release, several theoretical and (*semi*-)empirical models have been reported in literature. While for matrix systems (drug dispersed within a polymer network) the semi-empirical Korsmeyer-Peppas equation^51^ is commonly applied, this equation does not necessarily hold for reservoir systems such as the present one. For such systems, an analytic expression can be derived from Fick’s law of diffusion under the premise of perfect sink conditions, negligible (or just rapid initial) polymer swelling and that drug permeability does not change during the process^52^. While the time scales of polymer swelling are well below those of the dissolution process (minutes as compared to hours), it should be noted that *perfect* sink conditions might not be retained for all the conditions tested in the dissolution studies. The USP guidelines define sink conditions as “having a volume of medium at least three times the volume required to form a saturated solution of drug substance”^53^. Given the low solubility of indomethacin (see Methods and Supporting Information), this ratio is only about (3.0 ± 0.5) for the present system at 25 °C. While this represents the lower limit of perfect sink conditions, this should not impede the use of the (mathematical) model as solvent abundance (the key prerequisite) is still maintained. Also, increased indomethacin solubility at elevated temperatures means that perfect sink conditions are achieved for the release studies at 37 and 50 °C^54^.

For a slab geometry with a non-constant activity source^55^, the released drug fraction $\frac{{M}_{t}}{{M}_{\infty }}$ as a function of time *t* can be described by

4 $$\frac{{M}_{t}}{{M}_{\infty }}=1-{e}^{-\frac{A\cdot D\cdot P}{V\cdot L}t}.$$

Here, *A* denotes the total surface area available for release, *D* is the drug diffusion coefficient within the membrane, *P* is the drug partition coefficient between membrane and reservoir, *V* is the volume of the reservoir and *L* is the thickness of the coating. As only the diffusion coefficient is dependent on the polymer composition and the other parameters should not vary between samples (within the experimental limits), the exponent of Eq. (4) can be reduced to an *effective* release constant *k*:

5 $$\frac{{M}_{t}}{{M}_{\infty }}=1-{e}^{-k\cdot (t-{t}_{0})}$$

Introducing a time offset *t*_0_ as an additional fit parameter (*lag time*) greatly improved the data modelling, accounting for a dissolution onset different from the start of the experiment (i.e. insertion of the samples into the dissolution medium).

The model parameters fitted to the experimental dissolution data are summarized in Table 1 for all the samples investigated. In general, a good agreement between the mathematical model and the experimental data is found, as evident from *R*² values close to unity. The exception is the EGDMA homopolymer, which fits the theory the least. This is most evident for data collected at 25 °C, for which the fit yields an *R*² value of just 0.775. This underlines the previously discussed assumption that drug liberation in the case of the EGDMA homopolymer proceeds likely through imperfections in the polymer layer and is not due to diffusive transport as assumed by the model. It should be noted that similar results are also obtained when assuming an interfacial barrier model instead (e.g., the reaction-limited model described by Dokoumetzidis *et al*.^56^), as the mathematical description ultimately matches Eq. (5). A more detailed explanation is provided in the supporting information.

**Table 1:** Release constants *k* and time offsets *t*_0_ for the different samples at different temperatures as determined from non-linear regression of the experimental release data with the model presented in Eq. 5.

	T [°C]	*t*_0_ [min]	*k* [10^−3^ min^−1^]	*R*²
**No coating**				
*amorphous (as-prepared)*	25	0.39 ± 0.17	320 ± 5	0.978
*amorphous (heated)*	25	—	156 ± 5	0.985
*crystalline*	25	1.35 ± 0.14	38.9 ± 0.9	0.998
25% EGDMA	25	20.2 ± 1.4	2.0 ± 0.1	0.995
	37	5.75 ± 0.16	5.58 ± 0.04	0.988
	50	3.1 ± 0.5	21.5 ± 0.8	0.995
50% EGDMA	25	—	0.196 ± 0.002	0.995
	37	1.4 ± 0.4	0.388 ± 0.006	0.995
	50	—	1.19 ± 0.04	0.982
100% EGDMA	25	—	*0.030* ± *0.004*	*0.775*
	37	—	0.180 ± 0.005	0.986
	50	6.2 ± 1.7	3.0 ± 0.2	0.932

The parameter uncertainty is given as the (fit) standard error, *R²* is the coefficient of determination of the fit.

From the fit parameters in Table 1 it is also evident that the time offset *t*_0_ is mostly relevant for the samples covered by a 25% EGDMA coating. As these samples also exhibit the highest degree of swelling of the polymers tested, a causal connection might be suspected. At 25 °C, a time offset of approximately 20 minutes is determined, while equilibrium swelling is reached in about ten minutes (cf. Fig. 4a) For coatings of higher EGDMA content, this difference might not be observable as drug release proceeds much slower in general while equilibrium swelling does not take significantly longer.

The release constant *k* decreases by an order of magnitude between the different samples, with the uncoated samples showing the fastest release (*k* = (38.9 ± 0.9)10^−3^ min^−1^) and the EGDMA homopolymer the slowest (*k* = (0.030 ± 0.004)10^−3^ min^−1^) at 25 °C. Increasing the temperature to 37 °C doubles the release constant and an approximate fourfold increase is observed when dissolution is performed at 50 °C. While release constants are available at only three different temperatures, the data appear to follow an Arrhenius-like behavior. This is somewhat expected as, for example, temperature-dependent solvent diffusion in polymer systems is usually predicted by an Arrhenius term (Vrentas-Duda model)^57^. Anyway, the results indicate that different release behaviors are accessible by tuning the cross-linker fraction in the p(HEMA-*co*-EGDMA) polymer, so that coatings can be tailored to the therapeutic action required. It should be noted that without any or little cross-linking, a pHEMA polymer exhibits strong water uptake and detaches easily from the surface. This means such films are unstable under aqueous conditions, which might be undesirable for controlled release. The rapid removal of the coating layer exposes the drug layer directly to the dissolution media, and a rather immediate therapeutic action might follow, as indicted by the fast release observed in Fig. 7c.

In summary, different drug release behaviors were achieved by depositing iCVD polymers on top of thin crystalline films of indomethacin, with the release time frames spanning several orders of magnitude. The vapor-based polymer synthesis method allows for direct preparation of the coating atop the drug layer, ensuring a defined drug-polymer interface and minimizing the risk of any drug alteration often encountered in solution based processes. The p(HEMA-*co*-EGDMA) polymer coatings showed hydrogel formation when the cross-linker fraction is low, while little to no water permeation was observed for the hydrophobic EGDMA homopolymer. Likewise, drug liberation proceeded the fastest for coatings with low EGDMA content, with release rates being reduced as the EGDMA content increases. The ability to adjust the release behavior can enable individual therapeutic actions; short term pain relief might necessitate a large therapeutic input while chronical problems (e.g. rheumatic disorder) could make use of retarded drug liberation by means of small but continuous dosage, as provided from thin film patches.

## Methods

### Preparation of thin Indomethacin layers

Pharmaceutical grade indomethacin powder was purchased from Sigma-Aldrich (Germany) and used without further purification. Samples of indomethacin were prepared via drop casting on conventional microscopy glass slides (Roth, Germany). For this, indomethacin was dissolved in tetrahydrofuran (Sigma-Aldrich, Germany) at as solute concentration of 1 wt%, with the resulting solution being kept stirred prior usage. The substrates were cut to 2.5 × 2.5 cm^2^ pieces and cleaned in acetone and ethanol, respectively, and finally purged with a nitrogen stream. The substrates were carefully leveled before placing 200 µl of solution on the surface, which results in a homogenous drop spread with full surface coverage. After solvent evaporation, homogenous amorphous films are obtained. For the preparation of crystalline indomethacin films, amorphous samples were then exposed to an ethanol solvent vapor (solvent annealing^58^), which resulted in the entire film being transferred into a crystalline state within 24 hours.

### Preparation of drug coatings by initiated Chemical Vapor Deposition (iCVD)

Polymer coatings were prepared on the crystalline indomethacin samples by iCVD of the monomer 2-hydroxyethyl methacrylate (HEMA, purity 97%, Aldrich, Germany) and the cross-linker ethylene glycol dimethacrylate (EGDMA, purity 98%, Aldrich, Germany), using *tert*-butyl peroxide (TBPO, purity 98%, Aldrich, Germany) as initiator. For all the depositions, the substrate temperature was 28 ± 1 °C, the working pressure was 47 Pa and the filament temperature was 200 ± 5 °C. The TBPO flow rate was set to 0.80 sccm and a Nitrogen patch flow of 3 to 4 sccm was applied. By adjusting the monomer to cross-linker flow ratios, different polymer compositions were achieved (for compositional analysis, please refer to the supplementary information). The p(HEMA-*co*-EGDMA) polymers are labeled by their cross-linker volume fraction (EGDMA fraction), with 100% denoting the EGDMA homopolymer. For polymer characterization, pristine silicon wafers with a native oxide (Siegert Wafers, Germany) were coated in addition. For all samples, a nominal coating thickness of 200 nm was deposited (monitored *in situ* by laser interferometry).

### Polymer thickness characterization and swelling studies

Spectroscopic ellipsometric data were collected at three incidence angles (65, 70 and 75°) on a Woollam M-2000 ellipsometer (J.A. Woollam Co., USA) in the wavelength range between 370 and 1000 nm. For swelling experiments, the ellipsometer was equipped with a liquid cell attachment and measurements were performed in demineralized water at an incidence angle of 75°. Experimental data were fitted in the CompleteEASE^®^ software to a model consisting of bulk silicon, a native silicon oxide layer (1.7 nm) and a transparent Cauchy layer accounting for the polymer coating. In swelling experiments, the Cauchy layer is representing thickness and optical properties of the swollen polymer matrix and the ambient medium is set to water in the model.

### Morphological and structural investigation of Indomethacin films

Morphological characterization was performed on a standard AxioVert 40 ZEISS optical microscope equipped with polarizers. A FlexAFM atomic force microscope (Nanosurf, Switzerland), equipped with an Easyscan 2 controller, was used in non-contact mode using a TAP 300 cantilever (Budget sensors, Bulgaria). Data and image processing were performed using the freely available software package *Gwyddion*^59^. Crystallographic properties were probed on a PANalytical Empyrean reflectometer in *θ*/*θ* geometry (specular scans). The setup was equipped with a copper sealed tube (Cu Kα, wavelength λ = 0.154 nm), various slits and masks as well as a Pixel^3D^ detector. Data are presented in scattering vector notation which calculates as q_z_ = 4·π/λ·sin(θ).

### Thin film dissolution testing

Time-resolved drug release studies were performed in a non-standard dissolution experiment as the standard apparatus is unable to evaluate samples containing very low drug loads. Instead, an adapted system was employed. Indomethacin samples (on glass substrates) were placed in a cylindrical, sealed glass vessel containing 50 mL of 0.2 molar KH_2_PO_4_ phosphate buffer as dissolution media, adjusted to pH 5.8 with sodium hydroxide. The pH is slightly above the range of healthy and clean skin, representing more realistically the environment of potential patients’ skin^60^. Indomethacin solubility in phosphate buffer is reported in literature as 0.109 mg/ml (at pH 5.7)^46^. The indomethacin concentration in solution was probed at certain times by measuring the absorption at a wavelength of 321 nm with a nanophotometer (Implen, Germany). For this, 1 mL solution was taken from the vessel and placed in a quartz cuvette for the measurement. Thereafter, the solution was fed back to the system to keep the medium volume overall constant. The medium was not stirred during dissolution testing. Data points are given as the average of four samples, improving statistics and thus accounting for small differences in drug/polymer film quality during preparation.

### Data availability

Data available on request from the authors.

## Electronic supplementary material

Supporting Information
