# Supplementary material for: Controlling Indomethacin Release through Vapor-Phase Deposited Hydrogel Films by Adjusting the Cross-linker Density
Source: Sci Rep. 2018 May 8;8:7134. doi: 10.1038/s41598-018-24238-w (PMC5940858; doi:10.1038/s41598-018-24238-w)
Supplement: Supplementary file 1 — Supporting Information [file 41598_2018_24238_MOESM1_ESM.docx]

**SUPPORTING INFORMATION**

Controlling Indomethacin Release through Vapor-Phase Deposited Hydrogel Films by Adjusting the Cross-linker Density

Paul Christian,^†^ Stefan Tumphart,^§^ Heike M.A. Ehmann,^§^ Hans Riegler, ^§^ Anna Coclite^†^ and Oliver Werzer^§,*^

^†^Institute for Solid State Physics, NAWI Graz, Graz University of Technology, 8010 Graz, Austria

^§^Institute of Pharmaceutical Sciences, Department of Pharmaceutical Technology, University of Graz, 8010 Graz, Austria

* Corresponding authors: [oliver.werzer@uni-graz.at](mailto:oliver.werzer@uni-graz.at) (*main*); [paul.christian@tugraz.at](mailto:paul.christian@tugraz.at)

**AMORPHOUS INDOMETHACIN FILMS ON GLASS SUBSTRATES**


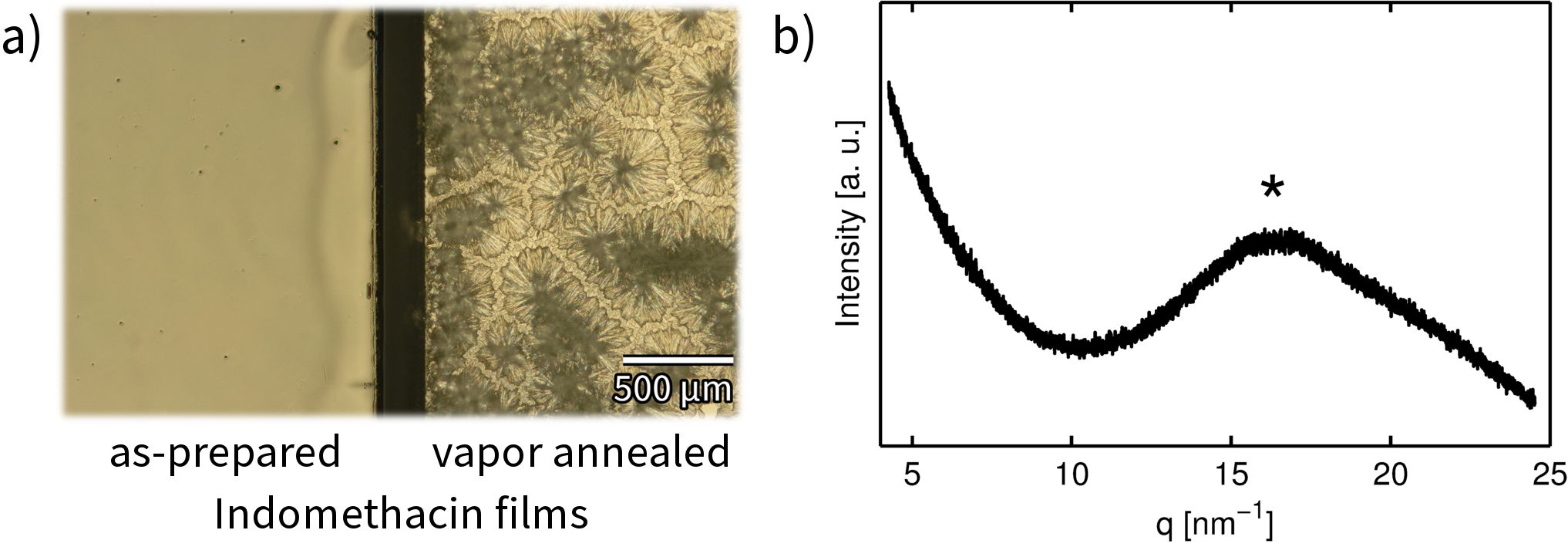


**Figure S1.** a) Optical microscopy image of drop cast Indomethacin films supported on glass substrates; as-prepared (amorphous) film (left) and crystalline sample (right) after ethanol solvent vapor annealing.
b) X-ray diffraction pattern of an as-prepared Indomethacin film, featuring an amorphous hump due to scattering from the glass substrate (marked by an asterisk). The absence of any diffraction peaks confirms the amorphous nature of the Indomethacin film.

**STRUCTURAL ANALYSIS OF SAMPLES COATED BY iCVD**


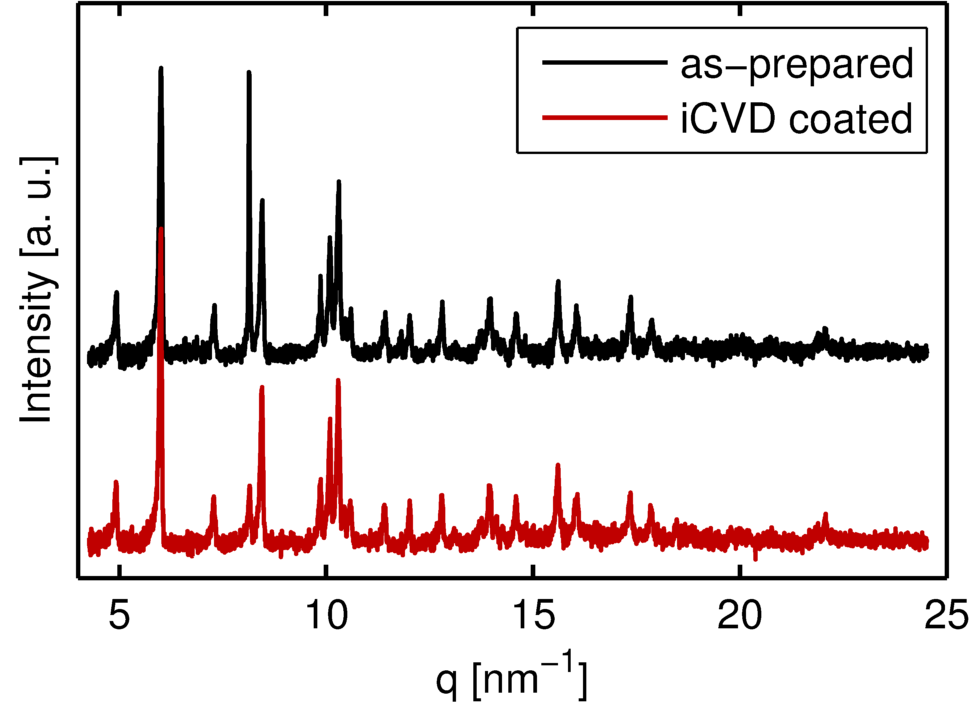


**Figure S2.** X-ray diffraction patterns of a crystalline Indomethacin film in the as-prepared state and after a 200 nm p(HEMA-*co*-EGDMA) coating was applied by iCVD. The data evidence that the Indomethacin crystal structure remains unchanged by the polymer coating. Please note that experimental data are baseline corrected to remove scattering from the amorphous glass substrate.

**POLYMER MORPHOLOGY ON SILICON**


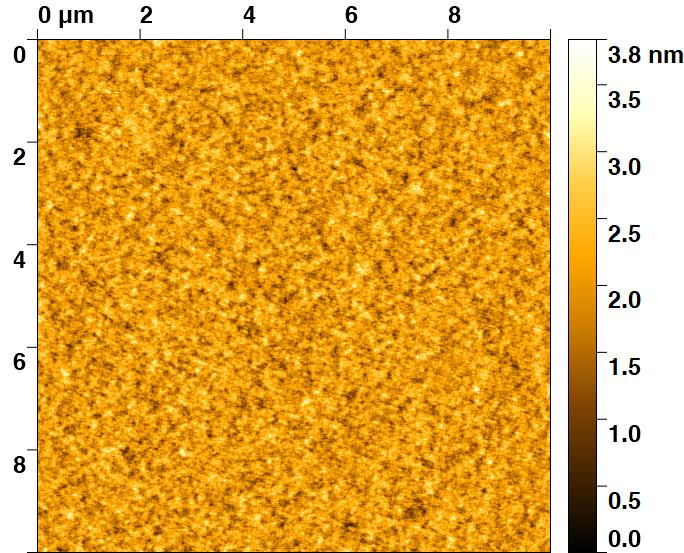


**Figure S3.** Atomic force micrograph of a p(HEMA-*co*-EGDMA) sample prepared on silicon. The EGDMA volume fraction is 25 %. The root mean squared roughness is 0.4 nm.

**INDOMETHACIN SOLUBILITY IN KH_2_PO_4_ BUFFER SOLUTION**

Indomethacin solubility at 25 °C was determined in a M 0.2 KH_2_PO_4_ buffer, adjusted to pH 5.8 with sodium hydroxide. For this, indomethacin was added to the phosphate buffer until a saturated solution was obtained. The solution was kept stirred for 24 hours. Subsequently, the solution was filtered and analyzed by UV spectroscopy. By comparing the absorption with a calibration curve obtained for diluted indomethacin solutions, an indomethacin solubility value of (0.098 ± 0.005) mg/mL was determined in the phosphate buffer.

**POLYMER COMPOSTIONAL ANALYSIS**

For the analysis of the polymer composition, iCVD polymers were deposited on single crystal silicon wafers. Fourier transform infrared spectroscopy (FT-IR) was then performed in transmission mode on a Bruker IFS 66v/s spectrometer. The experimental spectra were baseline correction by a custom routine written in *R*, utilizing routines provided in the *baseline* package^1^. The polymer composition was determined by treating the baseline-corrected experimental spectra as a linear combination of the constituting homopolymers, weighted by scaling factors (the fit parameters) for their respective volume fraction. A more detailed explanation of the applicability of this method in the analysis of thin polymer films is given elsewhere.^2^

In Figure S 2, the experimental spectra of two polymer compositions are provided. The compositional analysis reveals that the p(HEMA-*co*-EGDMA) polymers contain 25 % (a), respectively 50 % (b) EGDMA. The theoretical spectra from the fit are found in good agreement with the experimental data. The absence of a pronounced peak at 1620 cm^-1^ indicates that most vinyl bond are converted in the iCVD process (in the detection limit), i.e. the films are strongly crosslinked.


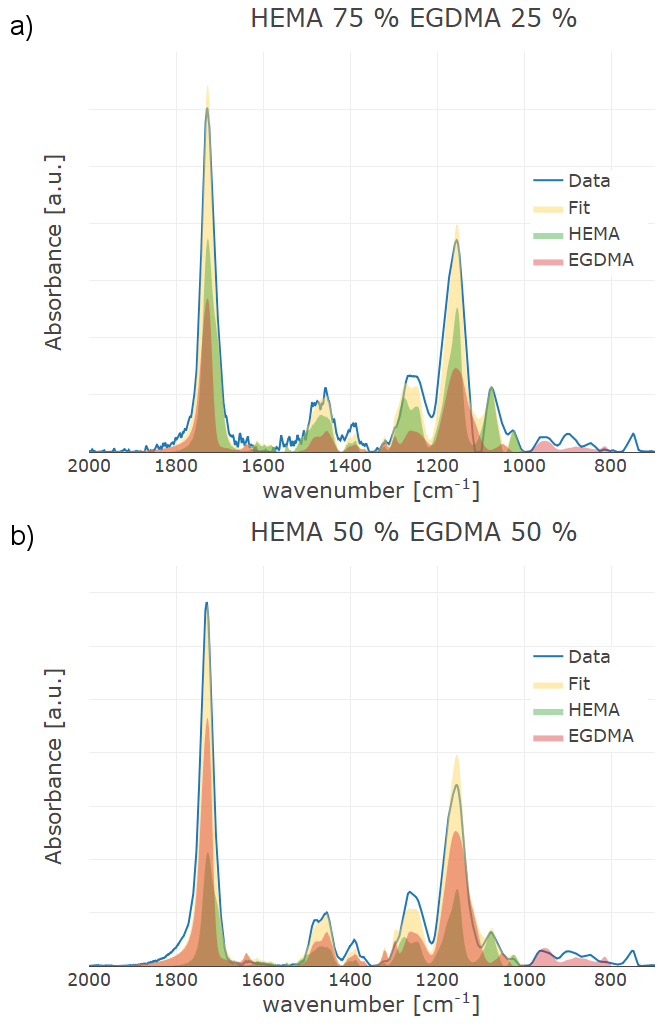


**Figure S4**. Exemplary analysis of the FT-IR spectra of two different iCVD polymers. The experimental spectra are evaluated as a linear combination of the homopolymer spectra of HEMA and EGDMA, respectively.

**SWELLING IN KH_2_PO_4_ BUFFER SOLUTION**


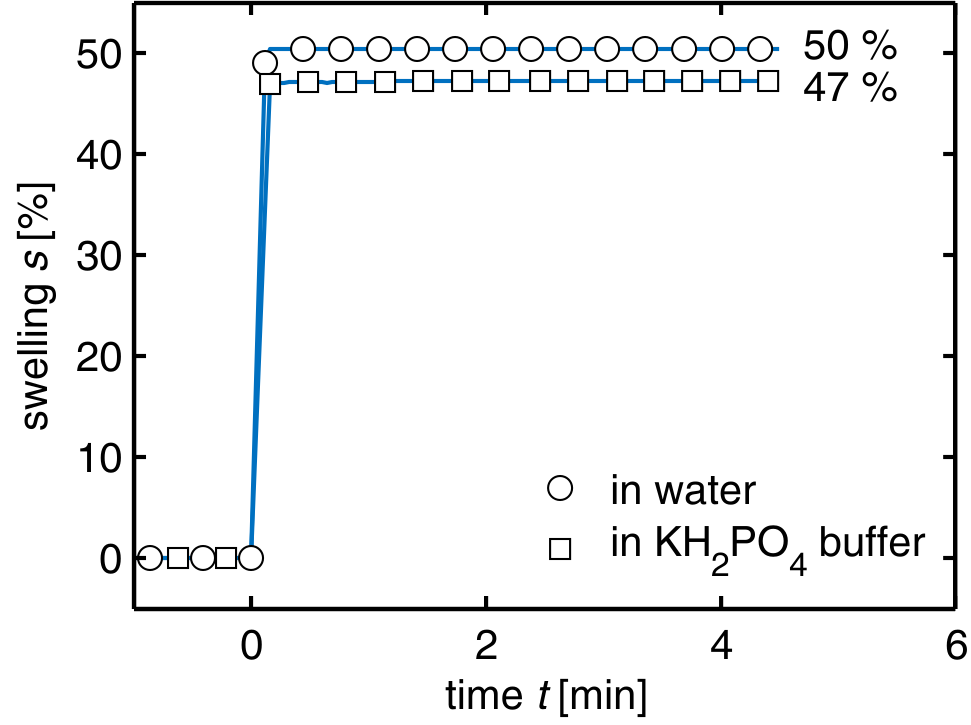


**Figure S5.** Swelling behaviors of a p(HEMA-*co*-EGDMA) film in water and in a 0.2 molar KH_2_PO_4_ buffer solution (adjusted to pH 5.8 with NaOH), as determined from ellipsometric measurements. Time *t* = 0 denotes the injection of the liquid media. Data modeling was performed according to the procedure outlined in the method section of the main text. To account for the difference between water and the buffer solution, optical constants were independently collected for the latter and then used as the ambient medium in the model.

**DISSOLUTION MODELING UTLIZING AN INTERFACIAL BARRIER MODEL**

According to the model developed by Dokoumetzidis et al., the change in dissolved drug concentration, $C$, can be described as a function of time $t$ via a bidirectional reaction equation of form^3^

|  | $\frac{dC}{dt}=k_{f}^{*}\left( \frac{D}{V}-C \right)^{a}-k_{r}C .$ | (S1) |
| --- | --- | --- |

Here,$D$ is the drug dose, $V$ is the solvent volume, exponent $a$ determines the order of the reaction and $k_{f}^{*}$ and $k_{r}$ are the reaction constants (the index *f* denotes a forward reaction, i.e. dissolution, while index *r* denotes the reverse reaction). This differential equation can be integrated numerically and fitted to the experimental data presented in the main text (see Figure S6). While the fits describe the experimental data reasonably well, they come with large parameter uncertainties (see fit results in Table S1). When evaluating Equation (2) for the homogenous case ($a=1$, assuming each undissolved species has the same probability of dissolving), an analytic expression can be found for the dissolved fraction $\phi$:^3^

|  | $\phi= \frac{k_{f}^{*}}{(k_{f}^{*}+k_{r}^{*})} \left( 1-exp(-\left( k_{f}^{*}+k_{r}^{*} \right)\cdot t) \right) .$ | (S2) |
| --- | --- | --- |

Also this model is describing the experimental data reasonably well (see Figure S6 and Table S2), but fit parameters exhibit much smaller confidence bounds. Moreover, most fits yield rather small values for the reaction constant $k_{r}$, so that ${k_{r}\ll k}_{f}^{*}$. However, for such cases Equation (S2) is corresponding (in form) to the diffusion model applied in the manuscript.


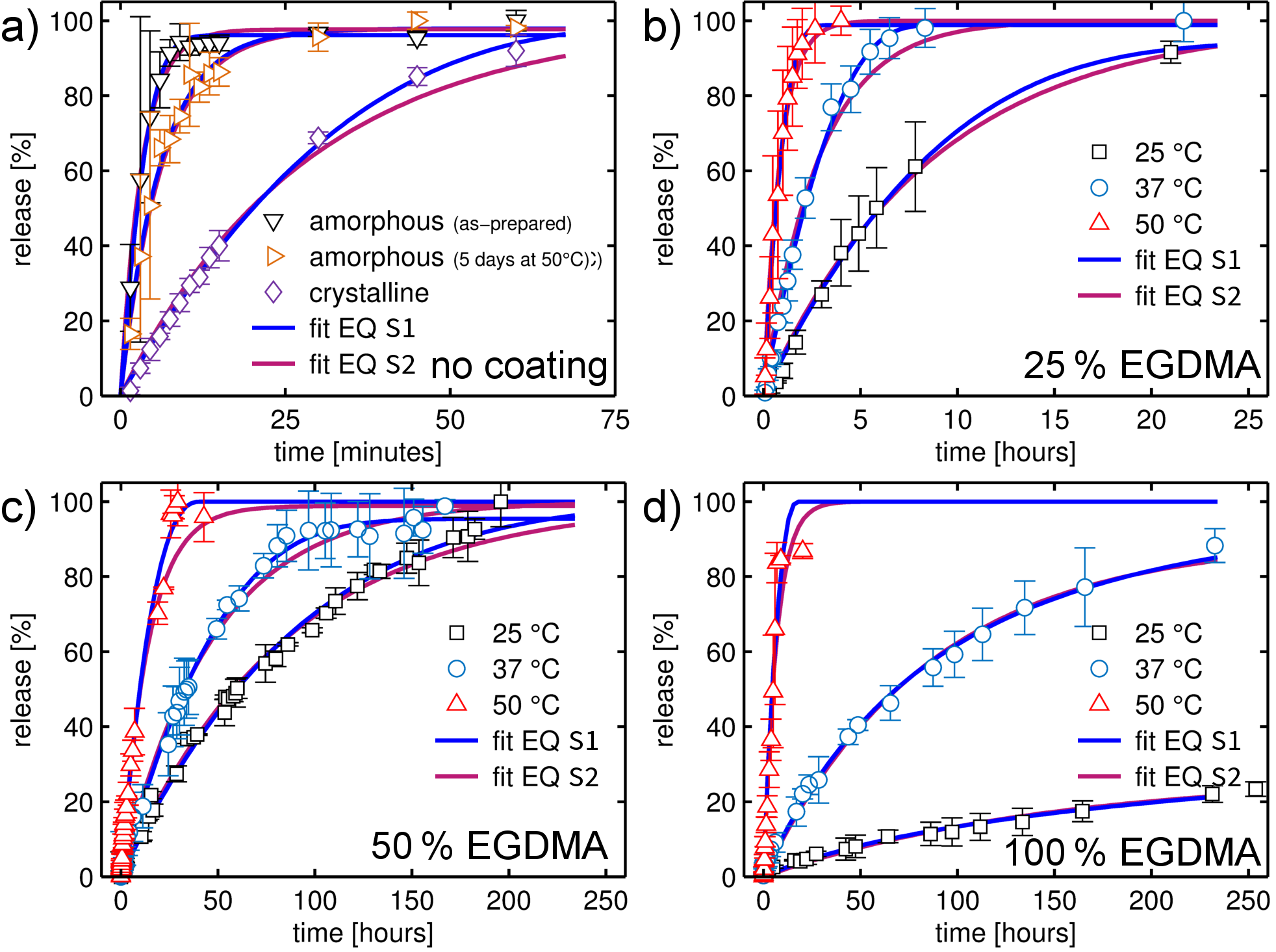


**Figure S6.** Fits to experimental dissolution data utilizing the reaction-limited model. The model is applied in its general form (Equation (S1), blue line) and for the homogenous case (Equation (S2), purple line). It should be noted that data were not weighted by their standard errors for this evaluation.

**Table S1.** Release constants$k_{f}^{*}$ and $k_{r}$ and exponent $a$ as determined from a fit of Equation (S1) to the experimental data presented in Figure S6. The parameter uncertainty is given as the (fit) standard error, R² is the coefficient of determination of the fit.

|  | T [°C] | $k_{f}^{*}$ [hour^-1^] | | | $k_{r}$ [hour^-1^] | | | $a$ | | | R² |
| --- | --- | --- | --- | --- | --- | --- | --- | --- | --- | --- | --- |
| **No coating** |  |  | | |  | | |  | | |  |
| *amorphous (as-prepared)* | 25 | 14.6 | ± | 0.9 | 4 | ± | 7 | 0.4 | ± | 0.5 | 0.604 |
| *amorphous (heated)* | 25 | 9.7 | ± | 0.8 | 0.2 | ± | 0.2 | 1.01 | ± | 0.15 | 0.863 |
| *crystalline* | 25 | 1.83 | ± | 0.05 | 0.14 | ± | 0.13 | 0.56 | ± | 0.11 | 0.993 |
| **25% EGDMA** | 25 | 0.106 | ± | 0.005 | 0.03 | ± | 1.4 | 0.4 | ± | 12 | 0.993 |
|  | 37 | 0.283 | ± | 0.010 | 0.05 | ± | 0.11 | 0.4 | ± | 0.4 | 0.997 |
|  | 50 | 0.94 | ± | 0.05 | 0.09 | ± | 0.15 | 0.5 | ± | 0.2 | 0.982 |
| **50% EGDMA** | 25 | 0.0110 | ± | 0.0005 | 0 | ± | 0.0012 | 0.8 | ± | 0.2 | 0.996 |
|  | 37 | 0.0191 | ± | 0.0013 | 0.01 | ± | 0.03 | 0.3 | ± | 1.1 | 0.998 |
|  | 50 | 0.062 | ± | 0.004 | 0 | ± | 0.007 | 0.61 | ± | 0.18 | 0.991 |
| **100% EGDMA** | 25 | 0.0023 | ± | 0.0004 | 0 | ± | 0.002 | 7 | ± | 4 | 0.977 |
|  | 37 | 0.0114 | ± | 0.0007 | 0 | ± | 0.0010 | 1.3 | ± | 0.3 | 0.996 |
|  | 50 | 0.13 | ± | 0.02 | 0 | ± | 0.05 | 0.57 | ± | 0.08 | 0.961 |

**Table S2.** Release constants $k_{f}^{*}$ and $k_{r}$ as determined from a fit of Equation (S2) to the experimental data presented in Figure S6. In this case, the release exponent $a$ is one for all the fits. The parameter uncertainty is given as the (fit) standard error, R² is the coefficient of determination of the fit.

|  | T [°C] | $k_{f}^{*}$ [hour^-1^] | | | $k_{r}$ [hour^-1^] | | | R² |
| --- | --- | --- | --- | --- | --- | --- | --- | --- |
| **No coating** |  |  | | |  | | |  |
| *amorphous (as-prepared)* | 25 | 18.0 | ± | 0.9 | 0.4 | ± | 0.2 | 0.991 |
| *amorphous (heated)* | 25 | 9.2 | ± | 0.2 | 0.20 | ± | 0.11 | 0.996 |
| *crystalline* | 25 | 2.10 | ± | 0.08 |  | 0 |  | 0.993 |
| **25% EGDMA** | 25 | 0.115 | ± | 0.004 |  | 0 |  | 0.992 |
|  | 37 | 0.351 | ± | 0.018 |  | 0 |  | 0.986 |
|  | 50 | 1.17 | ± | 0.05 |  | 0 |  | 0.989 |
| **50% EGDMA** | 25 | 0.0119 | ± | 0.0002 |  | 0 |  | 0.997 |
|  | 37 | 0.0213 | ± | 0.0003 |  | 0 |  | 0.997 |
|  | 50 | 0.069 | ± | 0.002 | 0.0008 | ± | 0.0015 | 0.997 |
| **100% EGDMA** | 25 | 0.0018 | ± | 0.0002 | 0.0050 | ± | 0.0010 | 0.968 |
|  | 37 | 0.0106 | ± | 0.0004 | 0.0012 | ± | 0.0004 | 0.994 |
|  | 50 | 0.148 | ± | 0.014 |  | 0 |  | 0.941 |

**SWELLING THEORY**

The derivation presented here follows the original work of Flory^4,5^, with the adaptations Toomey *et al*.^6^ introduced for the one-dimensional case.

The Gibbs free energy change upon swelling a system in a pure solvent can be described by two terms; the free energy of mixing $\Delta G_{mix}$ and the elastic free energy $\Delta G_{el}$, describing the expansion of the network structure.

|  | $\Delta G=\Delta G_{mix}+\Delta G_{el}$ | (S1) |
| --- | --- | --- |

According to the Flory-Huggins theory, the free energy of mixing can be described by

|  | $\Delta G_{mix}=RT\left( n_{1}\ln\left( \phi_{1} \right)+\chi n_{1}\phi_{2} \right) ,$ | (S2) |
| --- | --- | --- |

where $n_{1}$ denotes the *molar number* of solvent molecules occupying *volume* *fraction* $\phi_{1}$,$\phi_{2}$ is the polymer *volume* *fraction* and $\chi$ denotes the Flory-Huggins polymer-solvent interaction parameter. Note that this equation does not contain a term for the molar number of *polymer molecules* $n_{2}$, as a network structure does not include *single* molecules and thus $n_{2}$ is equated to zero.

For a linear deformation of factor $\alpha$, the free energy is described by

|  | $\Delta G_{el}=d\cdot RT\frac{\nu_{e}}{2}\left( \alpha^{2}-1-\ln\left( \alpha\right) \right) ,$ | (S3) |
| --- | --- | --- |

with $d$ corresponding to the number of dimensions in which swelling occurs and $\nu_{e}$ denoting the *effective* *molar number* of chains in the polymer network.

When swelling reaches the thermodynamic equilibrium, the change in Gibbs free energy becomes zero ($d\Delta G=0$), meaning that difference in chemical potential $\mu$ has to become zero as well:

|  | $\Delta G=\sum_{i}\mu_{i}dN_{i}=0$ | (S4) |
| --- | --- | --- |
|  | $\mu_{1}-\mu_{1}^{0}=\left( \frac{\partial\Delta G}{\partial n_{1}} \right)_{T,p}dN_{1}={dN}_{1}\left. \left[ \underset{I}{\underbrace{\left( \frac{\partial\Delta G_{mix}}{\partial n_{1}} \right)}}+\underset{\mathrm{II}}{\underbrace{\left( \frac{\partial\Delta G_{el}}{\partial\alpha} \right)\left( \frac{\partial\alpha}{\partial n_{1}} \right)}} \right] \right.=0$ | (S5) |

**Part I**

The polymer volume fraction $\phi_{2}$ is the ratio of initial volume $V_{0}$ to the swollen volume

|  | $\phi_{2}\left( n_{1} \right)=\frac{V_{0}}{V_{o}+v_{1}n_{1}} ,$ | (S6) |
| --- | --- | --- |

with $v_{1}$denoting the *molar volume* of the solvent. Therefore, evaluating part I of eqn. (S5) and utilizing $\phi_{1}+\phi_{2}=1$, one obtains

|  | $\left( \frac{\partial\Delta G_{mix}}{\partial n_{1}} \right)=RT\left[ \ln(1{-\phi}_{2})+\phi_{2}+\chi\phi_{2}^{2} \right] .$ | (S7) |
| --- | --- | --- |

**Part II**

Taking the derivative of $\Delta G_{el}$ with respect to $\alpha$, one obtains

|  | $\left( \frac{\partial\Delta G_{el}}{\partial\alpha} \right)=d\cdot RT\frac{\nu_{e}}{2}\left( 2\alpha-\frac{1}{\alpha} \right) .$ | (S8) |
| --- | --- | --- |

The partial derivative of $\alpha$ with respect to$n_{1}$ depends on the number of swelling dimension. For three-dimensional isotropic swelling, the inverse of the swollen volume fraction corresponds to the cube of the deformation factor, i.e. $\alpha_{3D}^{3}=\frac{1}{\phi_{2}}$ . For the one-dimensional case, $\alpha_{1D}=\frac{1}{\phi_{2}}$ accordingly. Together with eqn. (S6), the derivative reads

| *1D case* | $\left( \frac{\partial\alpha_{1D}}{\partial n_{1}} \right)= \frac{v_{1}}{V_{0}}.$ | (S9) |
| --- | --- | --- |
| *3D case* | $\left( \frac{\partial\alpha_{3D}}{\partial n_{1}} \right)=\frac{1}{3}\frac{v_{1}}{V_{0}}\alpha^{-2} .$ | (S10) |

**Thermodynamic equilibrium**

Evaluating eqn. (S5) with eqns. (S7)-(S10), one obtains

**1-Dimensional swelling**

|  | $0=dN_{1}\cdot RT\left[ \left. \ln(1{-\phi}_{2})+\phi_{2}+\chi\phi_{2}^{2} \right.+\frac{\nu_{e}}{2}\frac{v_{1}}{V_{0}}\left( 2\alpha_{1D}-\frac{1}{\alpha_{1D}} \right) \right]$ | (S11) |
| --- | --- | --- |

**3-Dimensional swelling**

|  | $0=dN_{1}\cdot RT\left[ \left. \ln\left( 1{-\phi}_{2} \right)+\phi_{2}+\chi\phi_{2}^{2} \right.+\frac{\nu_{e}}{2}\frac{v_{1}}{V_{0}}\left( 2\frac{1}{\alpha_{3D}}-\left( \frac{1}{\alpha_{1D}} \right)^{3} \right) \right] .$ | (S12) |
| --- | --- | --- |

Rearranging the equations and expressing $\alpha_{x}$ as function of $\phi_{2}$, one obtains

**1-Dimensional swelling**

|  | $\underline{M_{c}=-v_{1}\rho_{pol}\left[ \left. \frac{\left( \frac{1}{\phi_{2}}-\frac{\phi_{2}}{2} \right)}{\ln(1{-\phi}_{2})+\phi_{2}+\chi\phi_{2}^{2}} \right. \right]}$ | (S13) |
| --- | --- | --- |

**3-Dimensional swelling**

|  | $\underline{M_{c}=-v_{1}\rho_{pol}\left[ \left. \frac{\left( \phi_{2}^{\frac{1}{3}} -\frac{\phi_{2}}{2} \right)}{\ln\left( 1{-\phi}_{2} \right)+\phi_{2}+\chi\phi_{2}^{2}} \right. \right] ,}$ | (S14) |
| --- | --- | --- |

by introducing the average molecular weight between cross-links $M_{c}=\frac{V_{0}}{v_{e}}\rho_{pol}$, with $\rho_{pol}$ denoting the polymer density.

**REFERENCES**

1. Liland, K. H. 4S Peak Filling – baseline estimation by iterative mean suppression. *MethodsX* **2,** 135–140 (2015).

2. Tazreiter, M., Christian, P., Schennach, R., Grießer, T. & Maria Coclite, A. Simple method for the quantitative analysis of thin copolymer films on substrates by infrared spectroscopy using direct calibration. *Anal. Methods* (2017). doi:10.1039/C7AY01748K

3. Dokoumetzidis, A., Papadopoulou, V., Valsami, G. & Macheras, P. Development of a reaction-limited model of dissolution: Application to official dissolution tests experiments. *Int. J. Pharm.* **355,** 114–125 (2008).

4. Flory, P. J. Statistical Mechanics of Swelling of Network Structures. *J. Chem. Phys.* **18,** 108–111 (1950).

5. Flory, P. J. *Principles of Polymer Chemistry*. (Cornell University Press, 1953).

6. Toomey, R., Freidank, D. & Rühe, J. Swelling Behavior of Thin, Surface-Attached Polymer Networks. *Macromolecules* **37,** 882–887 (2004).
